# Human retrovirus promotes the plasticity of regulatory T cells into T helper type 1-like cells through the T-bet transcriptional activation in neuroinflammatory disease

**DOI:** 10.1186/ar3677

**Published:** 2012-02-09

**Authors:** Yoshihisa Yamano

**Affiliations:** 1 Department of Rare Diseases Research, Institute of Medical Science, St. Marianna University School of Medicine, Kawasaki, Japan

## 

Recently, it has become increasingly clear that some committed effecter and regulatory T (Treg) cells are not stable, and the plasticity of these T-cells may be related to the pathogenesis of autoimmunity and inflammatory diseases [1]. However, the precise mechanisms that allow for T cell plasticity have not yet been clearly understood. Human T-lymphotropic virus type 1 (HTLV-1) is a retrovirus that is associated with multiorgan inflammatorydisorders such as HTLV-1- associated myelopathy (HAM/TSP), HTLV-1- associated arthropathy (HAAP), uveitis, Sjögren syndrome, and polymyositis [2-5]. HTLV-1-infected T cells may contribute to development of these disorders, since the number of HTLV-1-infected T cells circulating in the peripheral blood is higher in patients [6]. HTLV-1 mainly infects CD4^+ ^T helper (Th) cells that play central roles in adaptive immune responses. Based on their functions, patterns of cytokine secretion, and expression of specific transcription factors and chemokine receptors, Th cells differentiated from naïve CD4^+ ^T-cells are classified into 4 major lineages: Th1, Th2, Th17, and T regulatory (Treg) cells. We recently demonstrated that CD4^+^CD25^+^CCR4^+ ^T cells, which mainly include suppressive T-cell subsets such as Treg and Th2 under healthy conditions, are the predominant viral reservoir of HTLV-1 in both adult T-cell leukemia/lymphoma (ATL) and HAM/TSP [7]. Interestingly, T-cells of this subset become Th1-like cells with overproduction of IFN-γ in HAM/TSP, suggesting that HTLV-1 may intracellularly induce Tcell plasticity from Treg to IFN-γ^+ ^T cells [7].In this study, using human T-cell line and HTLV-1 infected CD4^+^CD25^+^CCR4^+ ^T-cells of HAM/TSP patients, the virus-encoded transactivating HTLV-1 Tax protein was demonstrated to induce the IFN-γ production through the expression of T-box 21(Tbx21)/T-bet, a transcription factor that is known to direct the differentiation of naive CD4^+ ^cells into IFN-γ-expressing Th1 cell. HTLV-1 Tax was also demonstrated to enhance promoter activity of Tbx21/T-bet cooperatively with transcription factor Specificity Protein 1 (Sp1). Furthermore, transfer of HTLV-1 tax gene in CD4^+^CD25^+^CCR4^+ ^T-cells using a lentiviral vector resulted in the loss of regulatory function of these T cells. This is the first report to our knowledge demonstrating the role of a specific viral product (HTLV-1 Tax) on the expression of genes associated with T-cell differentiation resulting in plasticity of Treg cells into Th1-like cells. These results suggest that HTLV-1 infection-induced immune dysregulation may play an important role in the development and pathogenesis of HTLV-associated immunological diseasesthrough its interference in the equilibrium maintained among host immune responses.

## References

[B1] Zhou X Instability of the transcription factor Foxp3 leads to the generation of pathogenic memory T cells in vivo Nat Immunol 2009 10 1000 1007 10.1038/ni.1774 19633673 PMC2729804

[B2] Osame M HTLV-I associated myelopathy, a new clinical entity Lancet 1986 1 1031 1032 2871307 10.1016/s0140-6736(86)91298-5

[B3] Nishioka K Chronic inflammatory arthropathy associated with HTLV-I Lancet 1989 1 441 2563817 10.1016/s0140-6736(89)90038-x

[B4] Mochizuki M Uveitis associated with human T-cell lymphotropic virus type I Am J Ophthalmol 1992 114 123 129 1642286 10.1016/s0002-9394(14)73974-1

[B5] Nakagawa M HTLV-I-associated myelopathy: analysis of 213 patients based on clinical featuresand laboratory findings J Neurovirol 1995 1 50 61 10.3109/13550289509111010 9222342

[B6] Nagai M Analysis of HTLV-I proviral load in 202 HAM/TSP patients and 243 asymptomatic HTLV-I carriers: high proviral load strongly predisposes to HAM/TSP J Neurovirol 1998 4 586 593 10.3109/13550289809114225 10065900

[B7] Yamano Y Abnormally high levels of virus-infected IFN-gamma^+ ^CCR4^+ ^CD4^+ ^CD25^+ ^T cells in a retrovirus-associated neuroinflammatory disorder PLoS One 2009 4 e6517 10.1371/journal.pone.0006517 19654865 PMC2715877

